# Abdominal Fat and Muscle Measurement on Routine CT: Is It Useful?

**DOI:** 10.5334/jbr-btr.1038

**Published:** 2016-02-04

**Authors:** Maxime Nachit, Pierre Tréfois, Audrey Loumaye, Jean-Paul Thissen, Etienne Danse

**Affiliations:** 1 UCLouvain, BE

**Keywords:** Body composition, Sarcopenia, CT, L3, Fat, Muscle, Chemotherapy

Defining body composition has always been challenging and a wide range of methods have been developed. Among them, computed tomography (CT) is on the rise as a strong correlation exists between tissues at abdominal levels and the whole body composition [1].

Formulas link muscle and fat area at the third lumbar level (L3) with total fat mass and fat-free mass (mostly muscles and organs) in kilograms [2]. An index of muscularity (IMS) was also computed by dividing the muscle area at L3 by the height squared (cm²/m²).

This technique is mainly used in cancer studies. Imaging methods such as CT and magnetic resonance imaging (MRI) have shown that muscle and fat behave differently in cancer-related weight loss, part of the well-known cancer cachexia.

Here, we confirmed that classic tools such as body weight, body mass index (BMI) and body surface area (BSA) are not sufficient for evaluating body composition in a cohort of 152 colorectal and lung cancer patients. For instance, in normal BMI range (18.5–24.9kg/m²) and overweight/obese category (>25kg/m²), respectively 53% and 30% were sarcopenic.

Recent studies have shown that the role of muscle mass and quality is critical. Sarcopenia, used here as a synonym of low muscularity, is strongly linked to prognosis and dose-limiting toxicities of chemotherapy. That is consistent with different types of cancer and therapy [3].

Furthermore, these associations between CT-defined body composition and cancer outcomes were validated in more than 50 studies, including thousands of patients all over the world.

CT-based method takes advantage of images that are acquired in standard care for staging or follow-up of cancer patients. It is a prognosis tool that could also improve scaling of treatments. The field is promising and it is highly realistic for routine use in any hospital that has a computer and a CT.

## Competing Interests

The authors declare that they have no competing interests.
